# Beyond coverage: a qualitative study exploring the perceived impact of Gabon’s health insurance plan on access to and quality of prenatal care

**DOI:** 10.1186/s12913-020-05310-6

**Published:** 2020-05-30

**Authors:** N’doh Ashken Sanogo, Arone Wondwossen Fantaye, Sanni Yaya

**Affiliations:** 1 Interdisciplinary School of Health Sciences, University of Ottawa, Ottawa, ON Canada; 2 School of International Development and Global Studies, Faculty of Social Sciences, University of Ottawa, 120, University Private, Ottawa, ON K1N 6N5 Canada; 3 The George Institute for Global Health, University of Oxford, 75 George Street, Hayes House, Oxford OX1 2BQ, UK

**Keywords:** Universal health coverage, Antenatal, Quality of care, Gabon, Maternal health, Global Health

## Abstract

**Background:**

Access to affordable and adequate healthcare in a health system determines the universal health coverage achievement for all residents in a country. Achieving access to healthcare requires the availability of a financing system that ensures access to and provision of adequate care, regardless of the ability to pay. In sub-Saharan Africa, accessibility, use and coverage of prenatal visits are very low and poor, which reduces the quality of care. This paper explored the impact of a social health insurance scheme on the quality of antenatal care in Gabon.

**Methods:**

This qualitative study involved the analysis of data collected from semi-structured interviews and non-participant observations to assess the quality of antenatal care. The study elicited perceptions on the demand side (pregnant women) and the supply side (health professionals) in health facilities. Fifteen semi-structured interviews were conducted with pregnant women (aged between 15 and 49) and 5 with health professionals, who each had a seniority of at least 10 years, at different levels of care. Nine non-participant observations were also conducted. Coded transcripts were reviewed and analyzed using the Canadian Institute for Public Administration of Citizen-Centered Services model as an analytical guide.

**Results:**

On the demand side, women were generally satisfied with the prenatal services they receive in health facilities. However, complaints were made about the rudeness of some nurses, the high price of the delivery kit (50,000 XAF), and the fact that some essential medicines for maternity are not covered. On the supply side, participants agreed that compulsory health insurance is important in providing antenatal care access to those who need it the most. However, some problems remain. The participants outlined some logistical problems and a lack of medical equipment, including the stock of drugs, disinfectants, and the absence of clean water.

**Conclusion:**

Understanding the perceptions of pregnant women and health professionals regarding the quality of antenatal care can help to inform refinements to methods through which the services can be better provided. In addition, the study findings are vital to increasing the use of care, as well as combating high maternal mortality rates. Compulsory health insurance has improved the accessibility and utilization of healthcare services and has contributed to improved quality of care.

## Background

According to the World Health Organization (WHO), approximately 3.5 billion people in the world do not have full coverage of essential health services. At the same time, nearly 800 million people spend around 10% of their household income to pay for healthcare services [1]. Approximately 100 million people are in abject poverty because of financial payments made for healthcare**.** When out of pocket payments are required for health services, the poor are often not able to pay and receive required services [1]. Even the rich can experience financial risks due to severe or long-term illness.

To develop a sustainable solution to such an issue, in 2005, WHO member countries arrived at a common understanding on Universal Health Coverage (UHC) for their populations [2]. Universal Health Coverage is defined as a system that ensures people have access to quality care irrespective of the costs [1–3]. Developing countries have made Universal Health Care a priority in response to the Sustainable Development Goals (SDGs). To enhance and achieve UHC the WHO recommends prepaid health care services, through which catastrophic expenditures and user fees can be significantly reduced [2, 4, 5]. The move towards UHC requires the strengthening of health systems and strong financing models and structures, such as through a broad and equitable tax system where compulsory health insurance is implemented [6, 7].

To achieve UHC, many low-and middle-income countries (LMICs) have experimented with various funding and health financing reforms, such as national social health insurance [7]. Such schemes are essentially health financing tools that are significant for mobilizing resources and funds for health [8]. They are also financing tools for pooling risk, which spreads the risks of illness across a population, and ultimately improves the financial accessibility and overall utilization of health services, especially for the poor. In Indonesia, for example, the poor were the most likely to underutilize healthcare services and were less likely to be able to spend on health care relative to their needs [9]. India’s Vajpayee Arogyashree health insurance scheme improved health outcomes and reduced the financial burden of covered conditions [10].

In an effort to reach the Millennium Development Goals (MDGs), African countries have improved the overall health status of their population in the last 20 years [11]. The life expectancy at birth has risen from 46.2 years in 1991 to 55.3 years in 2017. Adult mortality has decreased by 24% between 1990 and 2018 from 361 to 280 deaths per 100,000 people. In addition, under-5 and maternal mortality rates have also declined by 54.2 and 40.7%, respectively [3, 11]. A declining number of infectious disease cases have also been witnessed. For example, HIV and malaria prevalence rates have declined by 57 and 42% respectively while mortality rates from tuberculosis have declined by 31% [3, 11].

The literature has also documented improvements to reproductive health and maternal outcomes, including declining rates of maternal mortality in the continent [3, 11]. However, these improvements vary across countries, with various discrepancies in access to adequate maternal care [3, 11].

Direct or out of pocket payments for healthcare services contribute to such disparities across the continent [11]. In fact, studies have shown that user fees provide limited access to health care for the poor [12–14].

While some countries are putting in place various initiatives to improve access to and coverage of health care services, others have only made commitments to UHC [2]. Within this context, Gabon has endeavoured through its political reform to create the National Fund for Health Insurance and Social Guarantee (NFHISG) to reach Universal Health Care [12–15]. As a result of the NFHISG, 78% of pregnant women have made at least 4 antenatal care (ANC) visits to the clinics [16]. The perceived quality of antenatal care services provided in health facilities can influenced utilization patterns of healthcare services for pregnant women [17]. The fund has influenced perceptions of the quality of healthcare services and thereby the utilization of antenatal care services. For this reason, it is increasingly necessary to assess whether health insurance has contributed to an increase in the perceived improved quality of antenatal health care in Gabon. Moreover, ever since the commencement of compulsory health insurance in Gabon 10 years ago, no study has assessed the quality of antenatal maternal care provided to the population.

Therefore, this paper explores the effect of Gabon’s Health Insurance Plan on the perceived quality of antenatal maternal care. Improved understanding of the perceived quality of antenatal care services in Gabon can inform policy and decision-makers on the implementation of programs that meet the needs of pregnant women.

## Methods

This study employs a qualitative approach (a combination of semi-structured interviews and non-participant observations) to assess the effectiveness of the insurance scheme based on the perceived quality of antenatal care. The study looked at the perceptions of indigents (households with an income equal to or less than 80,000 XAF (Central African CFA Franc) per month or 146 USD per month), who are beneficiaries of the compulsory health insurance of the NFHISG and its covered services. More specifically, we sought to a) understand the effectiveness of the insurance scheme program in improving access to antenatal care and b) identify gaps in design and implementation of NFHISG which limit its effectiveness.

### Conceptual framework

This study uses the Canadian Institute for Public Administration of Citizen-Centered Services model [18]. It is a multi-attribute model with five dimensions: speed, knowledge or skills of staff, courtesy or comfort, fairness of treatment, and results. These five dimensions (Table 1) simultaneously influence satisfaction [18].

**Table 1:** Description of the dimensions of the Erin Research Inc. model, 1998

Settings	Description
**Speed**	The reaction time to a request according to the expectations and needs of the customer. This is the time needed to obtain a service or a product. Speed requires: - That the time to get an appointment with professionals is reasonable; - Waiting time is short when you do not have an appointment; - The timeframe for obtaining the results of examinations or assessments is reasonable; - That the delay in obtaining diagnostic services (blood tests, x-rays, etc.) is reasonable.
**Courtesy or comfort**	Feeling of well-being resulting from a healthy, clean and orderly inventory of equipment perfectly adapted to different situations. This requires that: - The environment in the health facility is positive; - That the premises be built in a safe manner, such as safe stair ramps. Courtesy refers to politeness and good manners that make the customer feel comfortable.
**Knowledge or skills**	Appropriate and proven set of representations, skills and knowledge appropriately mobilized by a person or group in a work situation.
**Fairness of treatment**	The patient is satisfied with the way they were treated.
**Results**	The patient got what they needed.

### Research setting

This study is set in Gabon, a Central African country that straddles the equator. With a population of 1, 811,079 million, 87% of its population resides in urban areas [19]. The average age of the population is 26 years with half of the population being younger than 22 years. The education enrolment and literacy rates are high. Christianity is the predominantly practiced religion [19]. Though the country is considered as upper middle-income, human development indicators and outcomes are similar to countries with lower income. A third of its population lives in poverty [19].

### Participants

The study targeted pregnant women, as well as health professionals at different levels of care. The health professionals had seniority status for at least 10 years, and thereby ample experience in the provision of compulsory health insurance. Pregnant women were recruited at the Nzeng Ayong Health Center in Libreville. Regarding health professionals, the recruitment sites were a private room (an office connecting with the patient waiting room was allocated to us for the study) in the Okala Health Center, the CHUL (Centre Hospitalier Universitaire de Libreville), and the Jeanne Ebori Hospital. The four targeted health facilities are public institutions. Indeed, these institutions were chosen because the poorer populations tend to use the public institutions, whereas those who can afford private care tend to use private institutions. Therefore, the exclusion of private institutions does not affect the results since the study limited participants to poor women, which mainly use public institutions. Due to financial and time constraints, and the availability of participants, pregnant women were selected only at the Nzeng Ayong Health Center. Regarding health professionals, the diversity of sites is because the study sought different experts involved in the provision of pregnancy care. In Nzeng Ayong, women were interviewed but not health workers (they were really busy) while in other health centers, we were allowed to interview health workers but not women.

### Sampling

Participants were purposively sampled by reasoned choice, with an aim for maximum variation and diversification in the samples [15]. As mentioned earlier, pregnant participants had to be indigents covered by NFHISG. Pregnant women and health professionals were approached directly using a recruitment form and were presented the purpose of the solicitation and its voluntary nature. No participants refused to participate in the study. Pregnant women not covered by NFHISG were not included because they were more difficult to reach, as they rarely come to prenatal consultations. Also, we did not have the financial resources to select them outside the health centers.

Information saturation is the rule regarding the sample size of qualitative research [20]. The sample size varies depending on the type of information required, research purpose, stake, usefulness, credibility, effectiveness over time and the resources available. The purpose of the sample is to produce as much information as possible, regardless of whether the sample is small or large [21]. In other words, the study relied on the principle of information saturation based on the idea that there is a sufficiently large sample when the continuation of data collection no longer emanates new information.

### Research instruments

To understand the demographic factors, the primary author (NAS) collected data related to age, gender, educational attainment, marital status and number of children. NAS used guides that included open-ended questions to conduct semi-structured interviews. NAS used the questions asked to assess the quality of antenatal care by eliciting perceptions on both the demand (**see Additional** **file** **1**) and service supply sides (**see Additional** **file** **2**) in Libreville health facilities. Non-participant observations (**see Additional file** **3**) were conducted to analyze the interactions between beneficiaries and health workers during prenatal visits. The 5 dimensions of the Erin Research Inc. model discussed with participants during semi-structured interviews originate from a sample of issues:

- Speed of services at the health facility level (waiting time at reception, care service, laboratory, etc.)
- Relationship with the health staff (attentive listening, answers to questions asked)
- Quality of the services offered by the health staff (care, exams, medications)
- Equity in treatment and coverage and overall benefits of health insurance
- Strategies to help improve customer service

### Data collection and procedures

The interviewer (NAS) conducted 15 semi-structured interviews with pregnant women and 5 semi-structured interviews with health professionals from November 8 to 15, 2018. The interview guide was pilot tested with the key informants and necessary adjustments were made. The interviewer encouraged women and health professionals to speak about their experiences in order to elicit the interviewees’ perceptions and to identify the links established between their experiences [15]. Interviews lasted 45 min on average. All interviews were recorded using a Dictaphone and transcribed in verbatim, and accompanied by field notes taken during non-participant direct observations. The transcriber is a native speaker of the local language (French) and is also fluent in English. To identify the standard quality of care received by women, non-participant direct observations were conducted for 2 days [21, 22]. This method of observation consisted of analyzing the interactions between beneficiaries and health workers in various maternal health activities. The intent of this was to verify whether the care provided by health personnel met the five criteria of the Erin model that influence women’s perceptions of quality. This study made use of non-participant observations for triangulation with interviews. Triangulation is particularly important as one data collection method on its own is not usually reliable and use of multiple research methods for data collection help reveal insight about a specific topic [21].

The research interview is a limited, specialized, conversation-like interaction that shares many characteristics with informal verbal exchanges, except that it stimulates the situation where one of the parties is considered an expert and the conventions and the rules of conduct are imprecise [15]. The interviewer encourages the person to talk about what they know. In other words, it does not seek to shorten the conversation but rather to lengthen it to better grasp the way the participant defines the reality and finds links between the events [15]. The purpose of the interview was to know what the participant thought and to gather views that could not be directly observed, such as feelings, ideas, and intentions. The fundamental principle was to provide a framework within which respondents expressed their understanding of experiences in their own terms [15]. NAS and SY developed an interview guide for the women and the health professionals based on the five dimensions of the Erin Research Inc. model [18]. The model was designed to engage the participants and gather perceptions and opinions on antenatal care quality and by extension the strengths and weaknesses of NFHISG’s compulsory health insurance system.

### Data analysis

Audio-taped interviews and notes (for non-participant observations) were transcribed verbatim, and transcripts were compared with the recordings and notes for accuracy, respectively. After thoroughly reading through the transcripts multiple times and immersing into, and developing familiarization with, the data, initial topics and ideas relevant to the research questions arose. Content analysis was conducted on the NVivo 11 software. NAS and SY coded the data. Coding enabled a deconstruction of the data, sorting, isolation, classification with other similar content, and grouping of the data.

The authors carried out coding according to the interview guide. They read transcripts line-by-line and open coded. Notes and categories that described content were written on the margins of the transcripts. Secondly, NAS grouped notes and categories according to a coding scheme and created sub- categories. NAS also compared and contrasted sub-categories, with some merged into larger sub-categories with general content description. Then, the larger sub-categories with similar patterns, themes, content, understandings, trends, and incidences were grouped together to formulate main categories. Concurrently, the second rater (SY) independently analyzed the transcripts and coded the data to mitigate interpretative bias of a single researcher (NAS). The approach adopted was personal and the printed material noted key themes, concepts and ideas in the margins. These highlighted first and second order evidence interpretations supported the collected data. NAS met with the second rater to discuss coding decisions and ensure the credibility of the overall analytical process. This approach was used as a quality assurance mechanism to ensure inter-rater reliability (IRR) and trustworthiness of the study.

### Ethical approval

The protocol received ethical clearance from the University of Ottawa’s Office of Ethics and Research Integrity on November 26th, 2018 under the number H-08-18-876. There was no local institutional ethics committee at “Centre Hospitalier Universitaire de Libreville” (CHUL), but we received administrative consent (0001023/MSF/CHUL/DG) from the hospital officials and ensured that all necessary processes required were followed, and that all approvals were obtained prior to the commencement of the study. Given that this study involves primary data collection, the authors employed a rigorous ethical approach. All personal identifiers were removed from the respondent transcripts to ensure and enhance confidentiality. Participants were required to provide informed consent prior to participation.

## Results

Results are presented in two sections: 1) demand for services and supply of services through semi-structured individual interviews; 2) supply of services through non-participant direct observations.

### Characteristics of study participants

As displayed in Tables 2 and 3 below, 15 women and 5 health professionals participated in the study. For the educational level, overall, all of the respondents had secondary or higher education. Eight women interviewed had a secondary level of education, 7 had a higher level of education and all 5 health professionals also had a higher level of education. Though marital status was associated by study sites, in sum, a large proportion of respondents were currently married or living with partners (co-habiting). Of the 15 women, 6 were single and 9 were married or living with partners. Regarding the religious beliefs of respondents, Christianity was the religion practiced by all women. Of the 15 women, 11 were students and 3 were unemployed; 14 women had no income. Only 1 of 15 women had employment, but her monthly income was only about 80,000 XAF or 146 USD.

**Table 2:** Summary characteristics of women

Item	Study Site - Nzeng-Ayong
Male	0
Female	15
**Age**	
16–24	7
25–35	6
35 and more	2
**Education**	
No education	0
Primary	0
Secondary	8
Higher	7
**Marital status**	
Currently married/living with a partner	9
Not currently married	6
**Religion**	
Christianity	15
Islam	0
Traditionalist/ no religion	0
**Occupation**	
Business	1
Students	11
Unemployed	3

**Table 3:** Summary characteristics of health professionals

Item	Study sites		
	Okala	CHUL	Jeanne Ebori
Male	0	1	1
Female	2	1	0
**Age**			
16–24	0	0	0
25–35	0	0	0
35 and more	2	1	1
**Education**			
No education	0	0	0
Primary	0	0	0
Secondary	0	0	0
Higher	2	2	1

A total of 15 pregnant women aged 15–49 years of age in Nzeng-Ayong participated in the study. They were all educated (minimum end of high school) and all indigent (households earning less than 80,000 XAF per month or US $ 146). All the 15 women who participated in the study were covered by the NFHISG health insurance scheme. There were a total of 5 health professionals: 2 midwives in Okala; 1 midwife and 1 obstetrician gynecologist at the CHUL; 1 obstetrician gynecologist at Jeanne Ebori. All of the staff had at least 10 years of experience working with the NFHISG.

### Demand for services and supply of services through semi-structured individual interviews

The demand side results are detailed in the text below and are summarized in Table 4 below.

**Table 4:** Summary results according to the Erin Research Inc. model

Settings	Description
**Speed**	There is no problem regarding the acquisition of the NFHISG card because all the women interviewed said that they received the insurance card the same day of their application. However, the women complained about long wait times. They said they wait an average of 4 hours at the reception before being consulted by a health professional.
**Courtesy or comfort**	Essentially, the attitudes, behaviors and courtesy of the health professionals at Nzeng-Ayong were generally positive, except sometimes with nurses, who were said to display rudeness towards women. The “class of mothers” is an important innovation for women and helps a lot during pregnancy by giving them useful information about pregnancy and motherhood.
**Knowledge or skills**	The women unanimously agreed that the medical staff are competent at Nzeng-Ayong. Midwives are experienced and care about women’s well-being. However, clients alone cannot determine if professional health workers are knowledgeable and competent as they likely do not understand all the technical aspects of care. The non-participant observations of client-provider interactions should be another key data source for looking at this so that should be noted. Observations of client-provider interactions are generally considered to be the gold standard for measuring quality of care objectively.
**Fairness of treatment**	There is some inequity of treatment between insured women and those who pay for the consultation. Insured women wait longer than others before being received by the medical staff.

#### Speed

The main message conveyed by all the women who were interviewed at the Nzeng-Ayong Health Center is the fact that the waiting time for obtaining the NFHISG card - a health insurance card provided by the Gabonese government and which can be used whenever the patient goes to the health facilities - is reasonable. This is usually done the same day. However, the waiting time at the reception is really long because many women especially go to this health center due to the facility's reputation for providing high quality services.

*“To get my card it was really fast. I made the request on July 23, 2011 and I got it the same day in 30 minutes.” (Pregnant woman #3, 15–25 years old).*

*“Everything went fast. I got my card the same day. At the first ANC of my first pregnancy in 2012, I had no insurance. In the health center, there was a NFHISG service and the nurses directed me to this service. They made my card the same day, it was really fast.” (Pregnant woman #5, 15–25 years old).*

*“The services are fast. This center receives a lot of women. Did you see at the entrance? There are about forty women. A friend of mine advised me to come in the afternoon (even if they give you the appointment in the morning), it is better to come in the afternoon because at that time, the majority of women are gone and it goes more quickly. Before seeing the midwife, it is the nurse who welcomes us; this is my first visit here and I can say that I was well received.” (Pregnant woman #7, 25–35 years old).*

*“I arrived at 6am and left at 2pm. Here we wait a lot but we are better followed that is why many women come here. There are two midwives and it is not enough for all these women there.” (Pregnant woman #9, 15–25 years old).*

There is no problem regarding the acquisition of the NFHISG card because all the women interviewed said that they received the insurance card the same day of application. However, the women complained about long wait times. They said they wait an average of 4 hours at the reception before being consulted by a health professional.

#### Courtesy

All the pregnant women in Nzeng-Ayong were unanimous in their opinion that the interpersonal relationships with midwives are very good. On the other hand, pregnant women attending for ANC agreed that the nurses are sometimes rude.

*“They have been respectful to me, but I have witnessed insults at the reception to a woman because at her previous visit she was asked to do some exams that she did not have time to do and the nurse was rude to her.” (Pregnant woman #2, 15–25 years old).*

*“it’s okay, it’s okay, they’re respectful. Here it is first come first serve. Sometimes it happens that it is a woman turn to pass, it is called, she does not hear (does not answer). The nurses are obliged to call the next one. After when the woman who has been skipped realizes that her turn has passed, she complains and this can sometimes create tensions between the nurses and the women; but in general, there is respect.” (Pregnant woman #8, 25–35 years old).*

Also, all women agreed that they receive a lot of information on pregnancy, childbirth and the postpartum period during ANC visits. Indeed, there is the weekly class called “class of mothers”, which is a space for learning about pregnancy and motherhood.

*“I discovered a system for pregnant women and even those who have already given birth; the class of moms. Nurses talk to women to try to elucidate certain points because they have noticed that there are women who have problems, who do not get ready when they give birth. Sometimes it is the same day (the day of delivery) that they prepare their bags or look for money to buy the medicine kit.” (Pregnant woman #4, 30–40 years old).*

*“This is my first pregnancy, I saw my pregnant sisters and cousins but I did not know it was like that. At first, I did not have the NFHISG, it was painful, I took drugs, I vomited a lot and I really lost weight because I could not eat. Since I am insured and I can come, the midwife takes good care of me, they explain to me what to eat, how to prepare my bag and I attend Wednesdays and Fridays to the class of moms who is a space for learning about pregnancy and childbirth for us young people.” (Pregnant woman #6, 15–25 years old).*

Essentially, the attitudes, behaviors and courtesy of the health professionals at Nzeng-Ayong were generally positive, except sometimes with nurses, who were said to have poor interpersonal communication with pregnant women. The “class of mothers” is an important innovation for women and helps a lot during pregnancy by informing them of information about maternity.

#### Knowledge or skills

The women unanimously agreed that the medical staff are technically competent at Nzeng-Ayong. Midwives in particular are said to be experienced and caring about women’s well-being.

*“Really, I have nothing to complain about. For me it goes well, she takes all her time, she looks at food, ask questions, measure my stomach, take my weight. I’m satisfied. Look at all these women; they come here because the staff is competent.” (Pregnant woman #1, 15–25 years old).*

*“I am in my third pregnancy and I always come here because I know that I will be well followed. Midwives have experience and good advice. I also have my habits here so really there is nothing to add.” (Pregnant woman #10, 30–40 years old).*

Women are confident in this health center because it is renowned for the competence of its caregivers.

#### Fairness of treatment

The majority of the women interviewed told us of a surprising phenomenon. There is some inequity in treatment between insured women and those who pay for the consultation. That is, insured women wait longer than others before being received by the medical staff.

*“I did not have any problems. I have the NFHISG card and have always been treated the same as others. But women talk to each other and some complain that health professionals are privileging women who pay directly to women who have the card.” (Pregnant woman #12, 15–25 years old).*

*“There is discrimination. This is my third ANC. Here it is by order of arrival that you receive but I have often seen women who arrived after me received before me. We cannot complain because we are in need*.*” (Pregnant woman #14, 15–25 years old).*

## Results

All women agreed that the services offered and supported by NFHISG largely meet their needs expectations. Nevertheless, areas for improvement were identified. In order to improve their experience with the compulsory health insurance of the NFHISG, they would like some of the drugs needed during maternity to be covered by the NFHISG. They were also undivided in the claim that the 50,000 XAF delivery kit was too expensive for them and that the hospital was lacking certain key medicines.

*“NFHISG meets my expectations. Insurance helped me a lot to take care of me. For improvements, I think it would be nice if drugs that are not covered by the NFHISG and that are important during pregnancy were free. For example, the delivery order (drug kit) costs 55,000 XAF and it must be given before the delivery. Delivery is free but the delivery kit is not free. Also, each ANC costs 1000 XAF. It seems that it was 5000 XAF before but today with the NFHISG it costs 1000 XAF, it is expensive for the needy like us, normally it has to be free.” (Pregnant woman #11, 15–25 years old).*

*“The assurance of the NFHISG meets my expectations. Regarding the maternity I have to nothing to say, NFHISG does a lot for us. I’m happy but for example, NFHISG does not support my eye examinations. I had to change my glasses but I have to wait after my pregnancy because it is expensive (290,000 XAF). NFHISG supports the eyes consultation, but the purchase of the glasses is not considered. And then there are some drugs that are needed during pregnancy that are expensive but not supported as well.” (Pregnant woman #15, 25–35 years old).*

### Supply of services

NAS interviewed 2 midwives in the Okala health center, a midwive and an obstetrician gynecologist at the CHUL, and an obstetrician gynecologist at Jeanne Ebori Hospital. In total, 5 health professionals involved in maternal care were interviewed.

#### Health coverage

The main message conveyed by all those who were interviewed is the fact that the NFHISG health insurance is very beneficial for all parties as it enables the health professionals to provide antenatal care services for the indigents. The health professionals compared the current level of coverage with the old level of coverage, indicating that the current coverage provided by the social health insurance scheme is a positive evolution.

*“I can say that today it is a system that works well because when I arrived here 7 years ago at the CHUL, health care providers were not convinced by the NFHISG. I had the chance to attend a seminar while they were doing the launch of the NFHISG at the health center where I worked before, so we had the time to know the care sheet of the NFHISG, to know the prenatal checkup. But when I arrived here in 2011, the women were not even assured (out of 28 women there were only ten who benefited from the NFHIGS), but today, all Gabonese are covered and have access to care so the evolution is very significant.” (Health Professional #3).*

*“The mandatory health insurance of the NFHISG is a good thing as women who cannot afford treatment are covered up to 80%. Since April 2018 if I remember correctly, the coverage is now 100%. So, they do not pay anything.” (Health Professional #2).*

However, the health professionals pointed out that with regard to the available resources to provide maternity services, much remains to be done. At all the health facilities, there were logistical problems and insufficient supplies of medical equipment and drugs to care for patients. As a result, the provision of some maternal care services is inadequate and insufficient, reducing the quality of care.

*“We lack material especially in the delivery room. There are times when the delivery room is not well equipped, which leads us to ask for things to buy to the patients. The thread for the repair of perinates, betadine, syringes so it complicates the work because when presenting the prescription to the patient she wonders why she must pay for it while it is free but it is not our fault. At the ANC service level, there are fewer problems. However, the NFIGS should normally provide the tape to women but here we do not have one and they have to pay 1000 XAF. In addition, they must purchase their pregnancy check books that are not covered by the NFIGS.” (Health Professional #1).*

*“Regarding the equipment, we have evolved because at the beginning (in 2011 when I arrived here) we did not have an ultrasound system, we proceeded to the old way by listening in the belly of the woman but today we have several ultrasound machines. I can also say that our hospital is a reference hospital because we take the biggest cases here. We have women who have had a C-section, we have pathological pregnancies and in this kind of pregnancy we must take care of the fetal vitality. Today we have evolved in terms of equipment but there are still stockouts for disinfectants. We also lack bags to evacuate waste. By day, we can do 30 dressings, we put the waste in bags but since we do not have many, they last days here which is not very hygienic.” (Health Professional #3).*

*“The availability of materials and drugs in relation to demand is not sufficient. Hospitals do not have enough medicine and women have to go to the pharmacy themselves.” (Health Professional #5).*

Moreover, with regard to the workload, it appears from the interviews that it has increased significantly since the introduction of the NFHISG and that patients should respect the levels of care (first go to the health center before coming to referral hospitals). The workload is amplified by understaffing of health professionals in health facilities, both of which can reduce the quality of care.

*“The workload has changed since the CNAMGS was put in place but it is bearable. Here, the real problem is the lack of equipment and premises. If we each had our office, for example, we could receive many more women.” (Health Professional #1).*

*“Yes, the load has increased considerably. Many more women come for treatment. Levels of care are not respected. Women should first go to the health centers to do it again here, but out of habit and lack of information, they come here. We lack staff, we are understaffed, indeed I can do 30 bandages a day, the tongs must be washed, it is also necessary to register the patients (age, neighborhood, contact) to avoid the loss of seen and all this takes time. We do not have enough tongs, we made the request but since we wait. When I go home I do not even have the strength to cook cassava” (Health Professional #3).*

*“The charge has increased and everyone has heard that the structure has just reopened and that the facilities are new so everyone comes here. Even the one who live 10-15 km want to come here. We have a lot of work because women do not respect levels of care. I worked at the CHUL and I know that since we opened, we have a little unclogged the CHUL.” (Health Professional #5).*

#### Availability and accessibility of maternal and neonatal health services

In the different health facilities, the study found that the same package of routine care is offered and provided. However, at the Okala Health Center, availability and accessibility are not optimal as it has no operating room, which is normal for this level of care.

*“Here we have ANC, childbirth, postnatal counseling, vaccination, infant growth monitoring and family planning. I am also initiating a service (which I already initiated at Nzeng Ayong) of support and advice to women called the class of mothers. The class of mothers consists of a weekly grouping of one hour of women of the same term. For example, there is a special day for women in the first trimester, a special day for the second, a special day for the third, a special postnatal day, a special day for pregnant HIV-positive women.” (Health Professional #1).*

*“Here we do everything. We have the delivery room, operating room for caesareans and complications, maternity (postpartum diaper), neonatology for premature and intensive care, psychology service, social service, family planning, screening.” (Health Professional #4).*

*“We have all the services at the cutting edge of technology. ANC, natural childbirth, caesarean section, postnatal care, post abortion care. Abortion is illegal in Gabon but there are women who abort clandestinely and we are obliged here to take care of them.” (Health Professional #5).*

### Strengths and weaknesses

From all the interviews with health professionals from different health facilities, it emerged as a strength that the mandatory health insurance of the NFHISG has increased the accessibility and use of health services by women. Increased use of services reduces the risk for poor maternal outcomes, including morbidity and mortality. It is important to clarify that improvement in health outcomes is related to the quality of care received during health service contacts. So, increased attendance at health services needs to be combined with patients receiving evidence-based care to improve outcomes and quality of care can vary widely across facilities. The facilities included in our study are in the capital Libreville - one of which has a reputation for having high quality of care - and may not be representative of health facilities in the country as we noted earlier. As a weak point, health workers are unanimous in saying that there are delays in payment (from 6 months to 1 year) of the rebates they must receive for each covered patient who comes for consultation. This leads to a certain negligence of the patients who have the NFHISG. Accordingly, underpayment was another complaint and was a cause of poor motivation amongst health workers. The long wait times that were identified by the pregnant women were also corroborated by a health professional.

*“The strengths are that the NFHISG has given all groups of the population the opportunity to have access to the health center. In the past, I recorded a lot of untrained women who came only the delivery day, but with the NFHISG they are no longer afraid to come here. The first weak point is that we who work here we do not know exactly the return of the NFHISG that is to say that when a woman consults, I know that she has to pay a certain amount (co-payment) but at After a while, according to the texts I have a rebate. According to the texts, I must receive 25% of rebate but with the NFHISG, I do not know, I do the consultations but I am not paid. This is a really weak point which means that many colleagues are refractory to the NFHISG and neglect the patients who have the NFHISG. This is a complex phenomenon because the NFHISG is late in taking time to pay for the health center, which in turn takes time to pay the providers. The second weak point is that there are special pharmacies that take the NFHISG card. For women it is complicated because these pharmacies are not listed so that everyone goes to the pharmacy in the city center to be sure to benefit and it is sometimes far for the patients.” (Health Professional #1).*

*“The strong point is that we are reducing maternal and perinatal mortality because women have access to care. We are increasing the Gabonese economy because it’s free. Regarding the weak points, I would like the CHUL NFHISG services to be available 24/7 because sometimes women who are not covered (and who are eligible to be) arrive outside of their working hours (8:30am to 3:30pm). These women normally have access to services but since the service is closed, we are obliged to charge them. The other weakness is that claimant rebates arrive late. We work for free to treat the women covered and the government payments arrive from 6 months to 1 year later. This is not normal because there is a lot of work; it is demotivating because we make sacrifices that we make to work and to be far from our families.” (Health Professional #4).*

*“The highlight is obviously accessibility for all. The weak points really concern the management system. Normally a delivery costs 137000fcfa and on this amount I touch 37500fcfa. Per month, I can perform 100 deliveries which amounts to more than 3 million. With NFHISG, we do not see the color of money because women do not pay and we are paid about 6 months later and never the exact amount. Payments are around 600,000 and this is not normal, it really does not encourage providers. There have been strikes and complaints about that.” (Health Professional #5).*

### Use of available maternal and neonatal health services

All interviewees believed that the most used services are ANC, delivery, vaccination, and postpartum care. All women mentioned hospitality and waiting time as an important element in the use of maternal health services.

*“They use all services. They are numerous for the delivery, they are numerous in postpartum (the cesarized remain here at least 4 days), the service of vaccination is also very used.” (Health Professional #3).*

*“The factors that push women to use our services are of course reception, waiting times. Here, even if they wait a long time, they stay because they know that it is here that they will find the best care and that if there is a problem, we will refer them to reference hospitals.” (Health Professional #1).*

Regarding the quality of available infrastructure and equipment, health professionals agreed that there is a lack of drugs, equipment, and clean water in their facility. They are obliged every morning to fill and bring water containers to work. This is a major problem.

*“Here it’s clean, but some tools such as syringes and water are missing. There is an incredible lack of water here. It is always necessary to carry water in buckets or barrels for our work but also for the toilets of the patients for example.” (Health Professional #1).*

*“Thank God since the beginning of the year, we have an ultrasound system. But we are lacking disinfectants, bags to evacuate the waste of bandages and suppurations daily, sometimes we lack water … We have to fill a lot of water cans in advance.” (Health Professional #3).*

*“Here I have nothing to say because the infrastructure is all new and are at the cutting edge of technology. Look for yourself how beautiful it is and it smells new. The only problem is the lack of drugs.” (Health Professional #5).*

Finally, regarding the general quality of care, the health professionals agreed that it respects the standards with ongoing training of agents. However, in Okala, the midwife told us that some providers do not have the skills and capacity to perform their jobs.

*“I speak also with the Heart, so you have to exploit this well my son not to be badly taken. I have 30 years of experience and I can say that there is a problem of qualification of the health providers and that influences strongly on the quality. For example, during a family planning consultation, a poorly trained midwife prescribed the pill without knowing how to explain the side effects to the patient. If the side effects occur, the patient will simply stop the pill and the family planning consultation will not be used. The qualification of the staff also intervenes in the reception. Sometimes some midwives send back teenagers under the pretext that they cannot give birth in a low voice without taking the care to examine their pregnancy. All these problems come from lack of qualification. Our great advantage here is the cleanliness and our instrument of sterilization of the material.” (Health Professional #1).*

*“In Gabon here with the Jeanne Ebori Foundation, we are the reference. We are not perfect, we try to improve but I think it’s ok. I suggest doing supervisions more often. I am happy when my supervisor is there to supervise my work; it makes people responsible and hardworking. Also, I can say that, Libreville Medical School is the best in Central Africa. We have been trained by the best so unpretentious I can say we are good here.” (Health Professional #4).*

*“Here it is a reference hospital. The quality is really good, there are continuous staff trainings and quarterly assessments. In this aspect, there is nothing to say, we are good.” (Health Professional #5).*

### Supply of services through non-participant direct observations

The study conducted 9 non-participant observations in the Okala Health Center. All 9 non-participant observations were prenatal consultations with a midwife who has 30 years of experience. The midwife proceeded methodically and acted similarly for the 9 non-participant observations. It was apparent that she was following a protocol. She first looks at the notebook and takes the socio-demographic information. She builds rapport, puts women at ease, tells them about the mothers’ class, and discusses with them any possible pregnancy problems they may have while doing the medical examinations. She always ends these consultations by summarizing recommendations to the patient relative to their term and visit, making sure that all have understood. The main results of the combination of these 9 non-participant observations are presented below:

- Title of the health professional: midwife with 30 years of experience

- Speed

6:45 am, the midwife begins to settle in and calls the women in order of arrival. She systematically checks the notebook of all women once in the consultation room. For women who are at their first ANC, she takes socio-demographic information such as age, telephone number, and neighborhood. She also asks if they have an abortion history, and whether they have been tested for HIV and sickle cell disease.

- Courtesy, comfort

The midwife asks a patient who was absent at the mothers’ class why she was away. The patient was embarrassed, but the midwife comforted her, telling her that it was not a reprimand. She courteously told the patient that these sessions are very important to prepare for motherhood, especially at 8 months of pregnancy (which was the case for the patient). The patient promises to be present at the next session.

The patient does not really have a question. The midwife explains that she will examine her breasts and cervix while reassuring her. The midwife asks her if she has any special concerns. The patient answers no. The midwife asks her if she is taking her prophylaxis against malaria. The patient answers that yes, she still has a box, after which the midwife makes another prescription and makes an appointment for an ultrasound. She gives her a bottle and asks her to bring her urine to the next visit.

- Skills

The midwife asks if she has any particular problems with the pregnancy. The patient responds that she has stomach pain and hemorrhoids. The midwife prescribes Spasfon for the belly and Sphemifère for the hemorrhoids. She invites her to the next class of mothers while mentioning that in her notebook there is no information about her husband. She tells him about the screening test for sickle cell disease, and about HIV for her and her husband because they still have not done so, while asking her if she took these malaria drugs. She takes her weight and her pulse. She is about to make private contact during gynecological exams, so I leave the room.

All consultations were very similar and at the end, the midwife made a summary of the consultation by emphasizing the important points. The points included the followings:

- Prescription of drugs for childbirth,
- Mosquito net supply and prophylaxis against malaria
- Tests such as ultrasound, urine test, HIV test and sickle cell disease
- Importance of the ‘class of mothers’

- Fairness of treatment

The midwife proceeds in the same way for all the women observed with the same attention, the same attentiveness and the same procedures of care.

## Discussion

This study has explored and provided insight into pregnant women’s perceptions of the antenatal healthcare they receive and perceptions of health professionals regarding the services they provide at a few health facilities in Libreville. The findings demonstrated that women had positive experiences in terms of the services they are able to access and use in a health facility. Indeed, the waiting time for obtaining the NFHISG card is reasonable, as it is usually done the same day. Moreover, all women agree that they receive adequate information and advice about maternal requirements throughout the continuum of care. According to the women’s perceptions, the midwives were also commended for possessing the technical skills and competence required to provide adequate antenatal care. The health professionals also professed of the positive effect the NFHISG has had on their ability to provide care to women. However, some participants criticized the long waiting times and the inequity of treatment, with some health professionals accused of favoring and providing better care for privileged patients who pay out of pocket over those who have the NFHISG card. Other drawbacks reported were about the poor technical and interpersonal skills of some care-takers, the high price of the delivery kit (50,000 XAF), and the fact that some essential medicines for maternity are not covered. Both groups of participants also identified lack of resources, such as equipment, as an area of weakness and poor quality in the healthcare provision.

Several differences across various studies on quality measures, variation in the direction of the relationship and reliance on descriptive methods indicate that that there is no consensus among maternal health experts on the quality of care measures [23]. A systematic review of the effect of health insurance on use and provision of maternal health services, maternal and neonatal health outcomes suggests that health insurance could influence the volume and quality of maternal health services provided by affecting providers’ behavior [23]. Another systematic review on the determinants of women’s satisfaction with maternal health care found that improvements in the quality of care could improve the process of maternal care [24]. In particular, improvements in the interpersonal relationships between patient and provider is vital for patients to feel they are being treated respectfully and hospitably, regardless of sociocultural or economic context. The workload of health professionals is also an important element of quality, as it may affect the attitude and behavior of health professionals. Stress from workload and poor interpersonal skills were concepts that arose from the data, which highlights the need to reduce workplace stress in Gabonese health facilities. A study in Mauritania showed that the observed reduction in quality of maternal care was related to increased workload for the direct service providers. This was as a result of more insured patients being attended to while providers’ pay did not change [23, 25]. Greater attention should be paid to the attitudes and behaviours of maternal health care providers to improve maternal health, for the sake of both women and health care providers [26].

The findings from the interviews with the health professionals indicate that they all agree that the NFHISG health insurance is important for improving access to care for those who need it the most. However, some problems remain. They have all outlined as downsides some logistical problems and a lack of medical equipment, including the stock of drugs, disinfectants, as well as the absence of clean water. Stockout of caring equipment is also a major barrier to good quality maternal health care in Gabon.

The factors contributing to the stockout of medicines and equipment are related to lack of adequate capacity in manufacturing processes, industry consolidation, marketing practices, procurement and supply chain management (lack of electronic management systems), and poor medicine stock management [27, 28]. The acquisition of professional skills by health professionals to help know how to manage the use of equipment is also important [29]. An important downside of the NFHISG health insurance is that health professionals' claimant rebates arrive late (6 months to 1 year later) and always below the expected amount; this led to discrimination of patients with the NFHISG card.

A study by the WHO office in Gabon in 2018 found that late claimant rebates were due to funding malfunction of the NFHISG [30]. The population’s great adhesion to the NFHISG combined with the strike of the cell phone companies that finance the NFHISG was the main reason [31]. Cell phone companies went on strike because they did not agree with the initial proposition that they only pay for the health insurance of the indigent while many companies in the country had the same turnover [32]. This resulted in considerable delays in the payment of claimant rebates to health staff, and the demotivation of health professionals. According to research evidence, the motivation of caregivers is a critical component of the quality of care [30, 31], meaning it could potentially reduce the technical and/or interpersonal quality of care provision. To solve the problem of late payment, and for the sake of social justice, the Gabonese government has exempted the telephone companies. Since 2018, a tax of 1% (in addition to the Value Added Tax (VAT) of 18%) called Social Solidarity Contribution (SSC) is imposed on citizens on all purchases [30]. Funding sources for and indigent to date are thus constituted by the SSC, and the contributions of money transfer companies (Western Union, money gram, etc.) outside the Economic and Monetary Community of Central Africa (EMCCA) zone [30].

The interface between the demand and the offer of services can be observed through activities deemed important in obstetrical care, such as advice given to women regarding her pregnancy and her neonate. In this respect, both supply and demand side actors agree that the class of mothers is an innovative initiative that helps women a lot, especially those who are in their first pregnancy. In addition, the medical staff recommends that all women attend at least 4 antenatal visits as recommended by WHO and that they be reminded when they miss an appointment. This was the case in Nzeng-Ayong. Indeed, the use of another data source (non-participant observations) has allowed us to notice that the midwives in Nzeng-Ayong are competent and have good interpersonal communication with patients. Women are treated equitably in treatment and the midwives methodically follow a treatment protocol and emphasize the importance of antenatal visits and other steps during intrapartum.

This study shows that Gabon is on the right track in implementing its health insurance policy despite the fact that it has encountered funding problems. Moreover, some challenges remain. Health insurance eliminates financial barriers and increases the use of services, but it is important that these services are of high quality, and to do this, the patient care experience must meet their needs. This requires well-trained staff, a pleasant work environment, a tolerable workload, and the availability of drugs and equipment. With well thought out investments in human resources and accessibility issues, services that meet the needs of the population can be offered.

### Strengths and limitations

This study has two main strengths. First, it is the first study conducted in Gabon to assess the quality of antenatal healthcare after the introduction of the NFHISG. This type of study, by giving voice to women and service providers, is important for informing policy makers and politicians about the effectiveness of the policies they put in place and what areas need to be strengthened. The goal of a system is to provide access to quality care for each individual. Moreover, a great advantage of this study is the combination of interviews with observations. It enables objectivity and neutrality during observation and helps to identify needs in care quality by observing the work processes of health professionals. The triangulation of different sources of information (observations and interviews) adds to the rigor of our study.

However, findings from this study should be interpreted in light of several limitations. Women who are not indigent were excluded from this study because the primary focus of the Gabonese government on improving the maternal health of the poor. This is a limitation because the inclusion of women who are not indigent would have enabled comparison and provided counterfactual evidence. Due to the dominance of Christianity and the high level of education and literacy rates amongst Gabonese citizens in Libreville, this study was only able to include pregnant women who are Christians and have secondary school level of education or higher. This is a limitation as it prevents comparison with lower education levels and other religious affiliations. Moreover, the findings may not be entirely transferable to other contexts.

Due to time and availability, this study only recruited pregnant women from the health center of Nzeng Ayong. This limits the generalizability of the findings to other contexts, as women from other antenatal care providing health facilities may experience different realities than the women included in this study. The fact that women mainly attended Nzeng-Ayong Health Center because of its reputation and popularity for providing quality services does present a selection bias. This study design cannot draw conclusions on the effects of the NFHISG on ANC quality of care, if any. Other facilities which are less popular might not have seen many improvements in quality, which also limits the transferability of these findings to other contexts in Libreville and in other Gabonese communities. Moreover, this study only recruited pregnant women who came to the health center for ANC. Although our sample size was sufficient for this exploratory study, a more diverse sample would be informative.

The inclusion of women attending for childbirth services and postnatal care check-ups could have garnered a greater representation of the experiences of women throughout the continuum of maternal care and thereby the effect of the NFHISG. In the same vein, during non-participant observations, we did not have the chance to observe women in postnatal consultations. It is also possible that the Hawthorne effect took place during the non-participant observations of health professional-patient interactions, thereby limiting the true nature of their interactions. Overall, considering these limitations, any evidence of a link between health insurance and the perceived quality of care is not conclusive. An additional limitation that should be mentioned is the long recall period for women who reported on their experience obtaining their insurance card. Indeed, several were reporting on events that happened in 2011 and 2012 and our data collection was in 2018. Health workers were also asked to describe the situation when the insurance scheme was introduced in 2011, so their answers may be subject to recall bias as well given the long recall period.

While we have used multiple sources of data and approaches to analyzing data to enhance the credibility of the study, we could not access administrative data from the study facilities that could support some of our conclusions that access and use to services has increased over time among indigent women. That being said, this is the first exploratory study of its kind on the impact of a social health insurance scheme on the quality of antenatal care in Gabon.

## Conclusion

Understanding the perceptions of pregnant women and health professionals regarding the quality of antenatal care suggest some positive and negative effects of the introduction of the NFHISG on access to, use of and quality of maternal care services based on service users and health workers. Pregnant women agreed that they had a positive experience with the coverage and support provided by the NFHISG, while health professionals believed that the NFHISG improved their ability to provide quality care. However, waiting times for receiving antenatal services, inequity of treatment timing for insured and uninsured women, poor interpersonal communication with nurses, supply and equipment shortages were factors that diminished the effectiveness of the NFHISG and the corresponding quality of antenatal care. Therefore, in addition with the limitations of the study, the NFHISG cannot be said to be optimally effective, nor can its positive effects be correlated with the perceived high-quality services. Nevertheless, the scheme has been perceived to have had general success in improving the accessibility of care and improving certain components of care provision. As maternal and child health is a national priority in most countries, this study can serve as an example at the international level for developing countries in the implementation of their health insurance policy.

To achieve UHC, SDG 3.8 by 2030, and the Africa Agenda 2063, there are certain areas that require further improvements in Gabon. Strengthening of the Gabonese health system and training of care providers, including in interpersonal communication and the need to deploy more health workers to avoid huge workloads, is required [24, 26]. It is also recommended that national medical stores stakeholders be involved in drug and supplies management processes. These should specifically consider District health workers, who are the ultimate consumers of the supply chain [31]. The Gabonese government can implement a revolving drug fund system. This would be in form of “Special Pharmacies and drug stores” that will enhance availability of essential drugs in public facilities. All these will improve the quality of health care [31]. Significant investment in public human resources for health could be a solution to reduce the number of equipment stockout [32]. A decentralized healthcare system [33] as opposed to the conventional health care hubs can also be a solution to improve delivery of essential medicines and equipment [34]. For future studies, we recommend further research targeting indigent women who are not covered by the NFHISG and women who are not indigent in order to constitute comparison groups.

## Supplementary information

**Additional file 1.** Women Interview guide

**Additional file 2.** Health professionals Interview guide

**Additional file 3.** Framework for non-participant observation

## Data Availability

Data analysis is ongoing for subsequent publications. As a result, datasets generated and/or analyzed for this study are not publicly available but on request from the corresponding author.

## Abbreviations

UHC: Universal Health Coverage
SDGs: Sustainable Development Goals
NFHISG: National Fund for Health Insurance and Social Guarantee of Gabon
WHO: World Health Organization
SSC: Social Solidarity Contribution
EMCCA: Economic and Monetary Community of Central Africa
ANC: Antenatal care
IRR: Inter-rater reliability (IRR)
AF: Central African CFA Franc
CHUL: Centre Hospitalier Universitaire de Libreville
MDGs: Millenium Development Goals
VAT: Value Added Tax
