# Supplementary material for: Beyond coverage: a qualitative study exploring the perceived impact of Gabon’s health insurance plan on access to and quality of prenatal care
Source: BMC Health Serv Res. 2020 May 30;20:483. doi: 10.1186/s12913-020-05310-6 (PMC7260761; doi:10.1186/s12913-020-05310-6)
Supplement: Supplementary file 1 — Additional file 1. Women Interview guide [file 12913_2020_5310_MOESM1_ESM.docx]

**Women Interview guide**

Good morning, my name is XXX and I am pleased to speak with you today.

In this interview, I would like to discuss the following topics: the quality of maternal health care you receive, your health service expectations, and the factors that contribute to your satisfaction or dissatisfaction with maternal health services covered by NFHISG. With these topics in mind, can we start?

1. **Preliminary information**

Health Department:

Health center:

Age:

Marital status:

Household size:

Educational level:

Occupation:

Monthly income (approximate):

Date of joining the NFHISG:

Number of times he / she has used the services of NFHISG:

When did he / she use the services for the last time:

What was the approximate walking time to reach your health center:

1. **Erin research model: the 5 elements of satisfaction**

- **Speed**
- What was the elapsed time between joining the compulsory health insurance and obtaining the card?
- Does this delay seem reasonable to you? If no, how much time would you suggest?
- What do you think about the speed of services at the health facility level (waiting time at reception, care service, laboratory, etc.)?
- **Courtesy or comfort**
- How do you value your relationship with health staff (respect)?
- How do you rate the quality of the information provided by the health staff?
- How would you describe your exchanges with the health staff (attentive listening, answers to the questions asked)?
- During pregnancy, childbirth, or the postpartum period, do you have the possibility and authorization of the health staff to be accompanied by the person of your choice (especially during the labor and delivery period)?
- **Skills**
- How do you perceive the quality of the services offered to you by the health staff (care, exams, medications)?
- Fairness of treatment and preferences of beneficiaries
- Do you think that the beneficiaries of the NFHISG (whether rich or poor) are treated equitably?
- In your experience, do you think that the intimacy of women and newborns (during the perinatal period) is respected, and confidentiality assured?
- **Results**
- What are your expectations for the services offered by the NFHISG?
- Do the services offered meet your expectations?
- Does the NFHISG allowed you to take care of your health?
- What do you propose to improve the services offered by the NFHISG?
- What do you think are the main weaknesses of your experience with NFHISG?
