# Supplementary material for: Beyond coverage: a qualitative study exploring the perceived impact of Gabon’s health insurance plan on access to and quality of prenatal care
Source: BMC Health Serv Res. 2020 May 30;20:483. doi: 10.1186/s12913-020-05310-6 (PMC7260761; doi:10.1186/s12913-020-05310-6)
Supplement: Supplementary file 2 — Additional file 2. Health professionals Interview guide [file 12913_2020_5310_MOESM2_ESM.docx]

**Health professionals Interview guide**

Good morning, my name is XXX, I have the honor to speak with you today.

During this interview, I would like us to address the following topics: your views on the strengths and weaknesses of the NFHISG; the availability and accessibility of maternal and child health services covered by MFHISG; supply and demand of maternal and child health services

- **Preliminary information**
- Health Department:
- Health center:
- Function:
- Level of education:
- Years of experience in NFHISG’s services:
- Seniority in the health facility:
- **Health coverage**
- What do you think of NFHISG’s compulsory health insurance system?
- What do you think of the state of your available resources for maternal and child health services in relation to the demand?
- How do you rate your workload? Has it changed since the introduction of the NFHISG?
- **Availability and accessibility of maternal and neonatal health services**
- What are the maternal and neonatal services available at your health center? Probe: ANC, postnatal visits, delivery attended by trained personnel.
- Do you think that these types of services are adapted to the needs and expectations of the population?
- What do you think about the distance travelled by women to get to these services?
- What do you think of the financial costs of accessing these services (are user fees accessible to the poor)?
- What are the strengths and weaknesses of NFHISG's mandatory health insurance policy?
- What are the strengths and weaknesses of the maternal and newborn health input distribution network at your institution level?
- **Use of available maternal and neonatal health services**
- Can you tell us the types of services women use the most during pregnancy, childbirth or afterwards at your health center?
- What are the factors that push women to use some of these services?
- Why women did not use some of these services?
- What do you think of the current state of your infrastructures and equipment available to offer maternal and child health services to the population? (Maternity ward, block, post-natal care)
- What do you think of the quality of the care women receive in your institution?
