# Supplementary material for: Beyond coverage: a qualitative study exploring the perceived impact of Gabon’s health insurance plan on access to and quality of prenatal care
Source: BMC Health Serv Res. 2020 May 30;20:483. doi: 10.1186/s12913-020-05310-6 (PMC7260761; doi:10.1186/s12913-020-05310-6)
Supplement: Supplementary file 3 — Additional file 3. Framework for non-participant observation [file 12913_2020_5310_MOESM3_ESM.docx]

**Framework for non-participant observation**

| **Occupation** (nurse, doctor, midwife) | **Speed**  (waiting time at reception, care services, laboratory, etc.) | **Courtesy, comfort** (attentive listening, answers to questions) | **Skills** (care provided, availability of health care equipment and medicines) | **Fairness of treatment** (are all women treated the same?) | **Results** |
| --- | --- | --- | --- | --- | --- |
